# Dorsal Penile Nerve Block via Perineal Approach, an Alternative to a Caudal Block for Pediatric Circumcision: A Randomized Controlled Trial

**DOI:** 10.1155/2019/6875756

**Published:** 2019-03-27

**Authors:** Xiaocou Wang, Chaoxuan Dong, Deepti Beekoo, Xiaowei Qian, Jun Li, Wang-Ning Shang-Guan, Xuebin Jiang

**Affiliations:** ^1^Department of Anesthesiology, Critical Care and Pain Medicine, The Second Affiliated Hospital and Yuying Children's Hospital of Wenzhou Medical University, Wenzhou, Zhejiang 325027, China; ^2^Department of Anesthesiology, The First Affiliated Hospital of Jinan University and Guangzhou Oversea Chinese Hospital, Guangzhou, Guangdong 510630, China

## Abstract

**Background:**

General anesthesia combining with a caudal block (CB) has been commonly performed in pediatric patients undergoing circumcision surgeries. However, some severe complications have been suspected of a caudal block in the combined use. To avoid these issues of a caudal block, this study introduces a novel dorsal penile nerve block (DPNB) via perineum guided by ultrasound as an alternative to a caudal block in pediatric circumcision surgeries.

**Methods:**

A total of 104 pediatric patients scheduled for circumcision surgeries were involved and randomly divided into 2 groups: the CB group (n=52) and the DPNB group (n=52). A laryngeal mask was inserted followed by induction and maintenance anesthesia of inhaled sevoflurane. In the DPNB group, a dorsal penile nerve block (DPNB) guided by a real-time ultrasonography was performed by a single injection via perineum of 0.25% ropivacaine plus 0.8% lidocaine with total injection volume of 3-5ml. In the CB group, a dose of 0.5 ml/kg was given via the caudal canal following the same general anesthesia with that of Group DPNB. The time to the first analgesic demand after surgery is the key data collected for comparing between the two study groups. Heart rates and respiratory rates changes before and during the surgical procedure, pain score when leaving the PACU, and the time taken for the first micturition after a surgery were also recorded to analyze the differences in analgesic effects between the CB and DPNB groups.

**Results:**

No significant difference in heart rates and respiratory rates was found between the two groups before and during the surgery. Pain scores were similar before pediatric patients leave the PACU. However, the time taken for the first micturition after a surgery in Group DPNB is shorter than Group CB. The patients in Group DPNB asked for analgesics later than those in Group CB. Additionally, no significant differences in adverse effects were noted between two groups except the numbness of the lower limbs occurring less in Group DPNB.

**Conclusions:**

The ultrasound-guided dorsal penile nerve block via perineal approach can basically act as a safe and effective alternative to the caudal block in pediatric patients undergoing circumcision surgeries. Clinical Trials identifier is ChiCTR-IPR-15006670. Protocol is available at http://www.chictr.org.cn/showproj.aspx?proj=11319.

## 1. Introduction

The circumcision is one of the most common surgical procedures performed in pediatrics, with its rates variously ranging from 42 to 80% in the United States [1] and 7 to 10% for boys in the United Kingdom [2] and nearly 4% in China [3]. Many regional anesthesia techniques including the caudal epidural block[3], the classical dorsal penile nerve block (landmark-based [4] and ultrasound-guided [5]), the pudendal block [6], or even the lidocaine-prilocaine (EMLA®) cream [7] have been used for circumcision surgeries to minimize pain and complications.

A single dose caudal epidural block in conjunction with a general anesthesia has been reported to provide effective intraoperative and postoperative analgesia for circumcision surgical procedures [3, 8]. However, the use of a caudal epidural block for circumcision surgeries has been questioned due to some severe complications, such as urinary retention, delayed mobilization, and lower extremity numbness [7].

In this study, an ultrasound-guided dorsal penile nerve block via perineal approach for circumcision surgeries in children was reported recently [9]. This method is a potential alternative to the conjunctive method of a caudal block and general anesthesia. This clinical trial aims to evaluate the safety and the effectiveness of this new nerve block technique in the children undergoing circumcision surgeries.

## 2. Materials and Methods

Regulatory approval was given by the Hospital Ethics Committee of the Second Affiliated Hospital and Yuying Children's Hospital of Wenzhou Medical University (No. L-2015-02). This study was also registered in Chinese Clinical Trial Registry on June 28, 2015 (registration No.: ChiCTR-IPR-15006670, Principal investigator: Xiaowei Qian) and performed between July 2015 and May 2017 in the Second Affiliated Hospital and Yuying Children's Hospital of Wenzhou Medical University. Informed written consent was obtained from the parents or legal guardians of all pediatric patients participating in this study.

A total of 110 ASA physical statuses I to II boys, aged 7 to 14 years old with normal cognition who were scheduled for elective circumcision surgery under general anesthesia were included in this study. Exclusion criteria were (1) with any history of drug allergies; (2) complicated with coagulation dysfunctions; (3) parental refusals. The patients were randomly and evenly divided into two parallel groups, Group DPNB, and Group CB, with the random number generated by the Excel software (Microsoft Office, 2007 edition).

The patients received no premedication. Continuous noninvasive monitoring items including noninvasive blood pressure (NIBP), electrocardiograph (ECG), peripheral oxygen saturation (SPO_2_), and respiratory rate were obtained by the monitors (the IntelliVue MP50; Philips, Shanghai, China). General anesthesia was induced and maintained by inhalation of sevoflurane in oxygen mixed with air gas flow. A 22-G intravenous (i.v.) cannula was placed after induction. Spontaneous respiration was maintained via a selected laryngeal mask airway (Air-Q, Intubating Laryngeal Airway, Mercury Medical Co., Florida, US), and the inhaled sevoflurane was modified and maintained as 0.8 to 1.0MAC.

A caudal block was performed in the patients of Group CB with the lateral position followed by the loss of consciousness. A single injection of 0.25% ropivacaine (Naropina, AstraZeneca AB, Sweden) plus 0.8% lidocaine (Lidocaine Hydrochloride Injection, Shanghai Chaohui Pharmaceutical Group, China), a total of 0.5ml/kg, was administered using a standard anatomical landmark technique [10].

**Figure 1:**
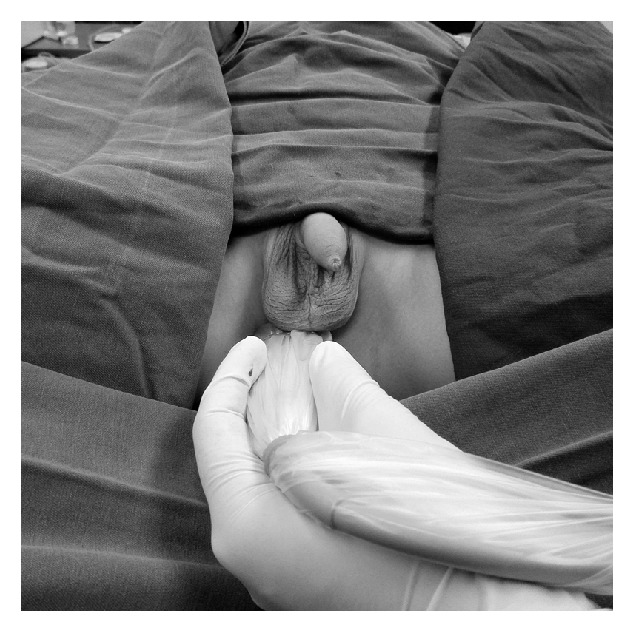
The position of the ultrasonic probe in the dorsal penile nerve block via perineal approach. For all patients of Group DPNB, a lithotomy position was adopted to fully expose the perineum. A linear array probe (5 to 10 MHz or 10-20 MHz) was used for an ultrasound guiding procedure. The probe was placed beneath the skin of the scrotum in a coronal parallel plane and scanned by a Sonosite M-Turbo (SonoSite, Bothell, WA, USA).

**Figure 2:**
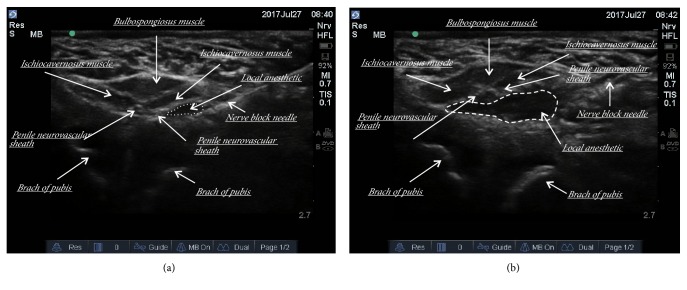
The ultrasound-guided dorsal penile nerve block via perineal approach. (a) an ultrasound image of a penile neurovascular sheath before a complete nerve block injection; (b) an ultrasound image of a penile neurovascular sheath after a complete DPNB injection. On the ultrasound image, injected local anesthetics were shown as a black hypoechoic area, firstly filled in one side of the neurovascular sheath and then spread to the opposite side.

The patients in Group DPNB received dorsal penile nerve blocks via perineal approach, under the direction of a real-time ultrasonography. A single injection of 0.25% ropivacaine plus 0.8% lidocaine, a total volume of 3-5ml, was given as detailed in our previous study [9]. A general anesthesia procedure was performed as described above. For all patients of Group DPNB, a lithotomy position was adopted in order to fully expose the perineum. A linear array probe (5 to 10 MHz or 10-20 MHz) covered by sterile slipcover was used for an ultrasound guiding procedure. The probe was placed beneath the skin of the scrotum in a coronal parallel plane (Figure 1) and scanned by a Sonosite M-Turbo (SonoSite, Bothell, WA, USA). The probe was adjusted and firmed until a paired penile neurovascular sheaths with arterial pulsing appear symmetrically in a short-axis view on the screen. The penile deep dorsal vein, the dorsal penile artery, the dorsal penile nerve, and a branch of the pudendal nerve lying inside the neurovascular sheaths are identified as oval-shaped or round structures. Under the real-time guidance of ultrasound, a needle was inserted and slowly advanced until approach the penile neurovascular sheath. After negative aspiration, local anesthetics were administered by a single injection. On an ultrasound image, injected local anesthetics were shown as a black hypoechoic area, firstly filled in one side of the neurovascular sheath and then spread to the opposite side (Figure 2). If the opposite side of the neurovascular sheath was not filled with local anesthetics fully, an additional 2ml of local anesthetic was given by relocating the needle the opposite side of the neurovascular sheath. Caudal blocks and dorsal penile nerve blocks were performed by well-experienced pediatric anesthesiologists blinded to the study. The surgical stimulus was applied more than 15 minutes after nerve blocks. All circumcisions were performed using the same surgical technique by senior pediatric surgeons. All information on surgeries was recorded.

Before and during surgical procedures, five specific time points were set up to record and compare heart rates and respiratory rates: T1, the time before any anesthesia procedure; T2, the time on inserting a laryngeal mask; T3, the time on removing the prepuce; T4, the time on stitching the last part of prepuce; T5, the time on pulling out the laryngeal mask. Remedial analgesic measures are determined by the duty anesthesiologist.

All patients undergoing surgical procedures were observed in the postanesthesia care unit (PACU) until all life signs are back to the normal before they were sent back to the surgical ward. Before leaving the PACU, all patients were evaluated on the postoperative pain score using two pain rating scales by an anesthesia nurse blinded to this trial. The Numerical Rating Scale (NRS) was used for pain evaluation (NRS, rating from 0 to 10. 0 for “no pain at all” and 10 for “worst imaginable pain.”). The stay time in PACU, the time on the first micturition, and the time on the first analgesic demanded by their parents or themselves were also recorded. Adverse effects such as nausea, vomiting, numbness of the lower limbs, and other postoperative complications within the 2 days after the surgeries were also recorded and compared.

## 3. Statistical Analysis

The sample size was calculated based on the result of our pilot study using commercially purchased software (PASS for Windows version 11.0; NCSS Inc, Kaysville, Utah USA). A study including 88 patients (n=44) would have the power (90%) to detect significant differences in the time at the first analgesic demand by the children between Group DPNB and Group CB. In consideration of dropping-out, we chose to enroll 52 patients per group (a total of 104 patients).

All data was analyzed using commercially purchased statistical software (SPSS for Windows version 13.0; SPSS Inc., Chicago, IL, USA). Student's* t-*test was used to examine group age, weight, height, and body mass index (BMI). Data at different time points of heart rates and respiratory rates were analyzed by variance analysis of repeated measures. Student's* t*-test was adopted to compare groups for the durations of surgery and PACU. The Mann-Whitney* U* test was used to examine pain scores, the time to first micturition, and the time at the first analgesic demand in groups. The quantitative datasets that were expressed as frequency or rate were compared using a Chi-Square test or a Fisher's exact test. The calculated* P* value was less than 0.05 as a statistical significance.

## 4. Results

**Figure 3:**
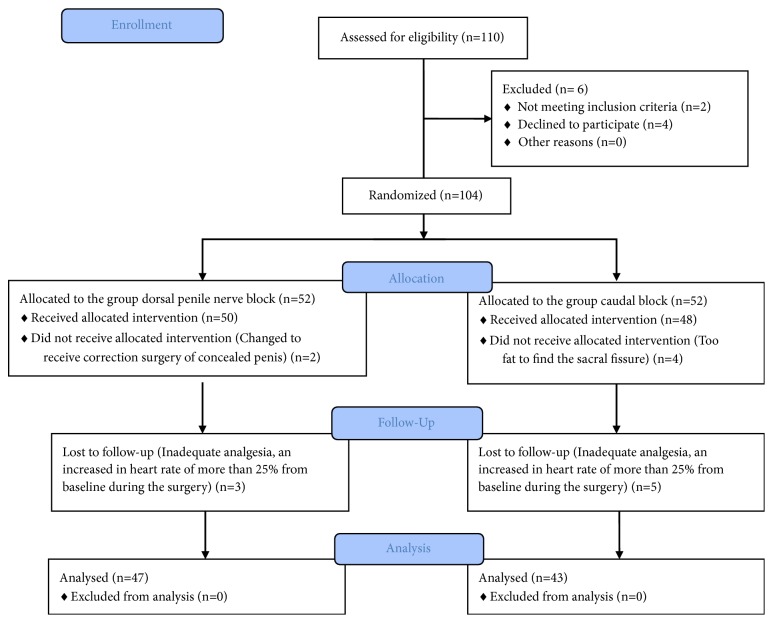
A CONSORT flow diagram of this study.

A total of 104 patients were enrolled. With 14 dropouts, 90 patients were recruited and finally analyzed, with 47 in Group DPNB and 43 in Group CB (Figure 3). All patients completed surgeries under general anesthesia with a caudal block or a dorsal penile nerve block. No severe complication occurred in this study. The characteristics of the patients are presented in Table 1. No difference was found in the mean age, weight, height, and BMI between the two groups (all* P* values were more than 0.05). Neither positive blood of initial needle aspiration nor hematomas was found during and after circumcision in Group DPNB. No significant difference in the duration of surgery or the stay time in PACU was detected, as shown in Table 2.

**Figure 4:**
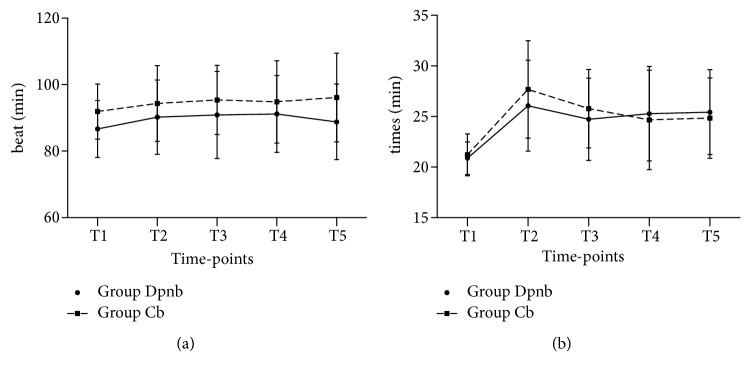
Changes in heart rates and respiratory rates before and during the surgical procedures between the two groups: Group DPNB, the dorsal penile nerve block, and Group CB, the caudal block. Image (a) heart rates; Image (b) respiratory rates. T1, the time before any anesthesia procedure baseline (the data collected from the medical records); T2, the time-point after on inserting a laryngeal mask; T3, the time on removing when the prepuce is removed; T4, when the time on stitching the last part of prepuce; T5, time-point the time after on pulling out the laryngeal mask. A* P* value less than 0.05 is considered as a statistical significance. No significant change in HRs and RRs between the two groups is found.

No significant difference in heart rates or respiratory rates changes was noted between the two groups at all five timepoints (all* P* values are more than 0.05), presented as Figure 4. Also, there was no significant difference in pain score when patients leaving the PACU between the two groups (Table 2). However, the time taken to first micturition after surgeries in Group DPNB is shorter than Group CB (131.3±21.1min in Group DPNB versus 290.5±43.9 min in Group CB,* P*<0.01), and the patients in Group DPNB asked for the first analgesic later than those in CB (262.1±43.1 min in Group DPNB versus 174.3±20.5 min in Group CB,* P* <0.01). Nine boys of 47 in Group DPNB received additional injection of 2ml of local anesthetic in the opposite side of the neurovascular sheath.

No statistically significant difference in the incidence of postoperative nausea or vomiting was found in the two groups (*P* value = 0.28). However, 4 patients in Group CB suffered from postoperative numbness of the lower limbs while no patient was observed numbness of the lower limbs in Group DPNB (*P* < 0.01).

## 5. Discussion

This study aims to explore the effectiveness and the safety of ultrasound-guided dorsal penile nerve block via perineal approach for children undergoing circumcision surgeries. Results show that an ultrasound-guided DPNB via perineal approach is similar to the CB technique at the safety and the effectiveness, which can serve as an alternative to the caudal block in circumcision surgeries in pediatric patients. The ultrasound-guided DPNB via perineal approach also provides some advantages at longer analgesia time, less incidence of numbness of the lower limbs, compared to the caudal block.

The caudal block [11], a mature nerve block technique, is the most common regional anesthesia performed in children undergoing circumcision. In this study, patients in Group CB received adequate analgesia during and after circumcision, and the first time call for analgesic demand is nearly 3 hours after surgery, which is consistent with other similar studies [6, 8, 11]. However, the disadvantages of caudal block such as higher incidence of urinary retention, motor block, and numbness of the lower limbs still need to be addressed and also exist in this study. Additionally, recent evidence shows that the caudal block will increase the occurrence of urethrocutaneous fistula in the hypospadias repair, compared to the penile block [12, 13].

The dorsal nerve of penile is one of the major terminal branches of the pudendal nerve [14], which is accompanied by branches of the internal pudendal artery (dorsal penile artery). The sensation of the penis is innervated by the dorsal nerve of penile. Therefore, a dorsal penile nerve block may be a better choice for children undergoing circumcision surgeries than a CB, by avoiding the adverse effects occurring in the CB. However, the dorsal penile nerve block has its anatomical limitation so that ultrasound guidance is necessary in the application of the technique. The dorsal penile nerve is a thin terminal nerve and is located in the perineum tissue deeply. All these properties determine the difficulty of the use of the DPNB. Additionally, the penile neurovascular sheath accompanying the dorsal artery of penile can be found by arterial pulse under ultrasound guidance and the dorsal penile nerve block can be achieved by injection of local anesthetic into the neurovascular sheath. Based on the results of this study, an ultrasound-guided DPNB can provide an appropriate analgesia without significant adverse event in those pediatric patients. Therefore, an ultrasound-guided dorsal penile nerve block via perineal approach was a reliable and feasible technique in children undergoing circumcision surgeries.

The duration of peripheral nerve blocks is one of the main indicators evaluating the effectiveness of nerve blocks. In this study, the time to first analgesic demand after surgery is defined as the primary outcome. Literatures reported a great variety of different times to first analgesic demand in the caudal block after circumcision surgical procedures [10, 15]. Sandeman et al. [15] reported the first analgesia time is 179±89 min with 0.2% ropivacaine 1 ml/kg alone or mixed with clonidine 1 *μ*g/kg by the caudal block. Taylor and colleagues [10] found that 0.25% levobupivacaine administered as a caudal injection at a dose of 2 mg/kg could persist 7.95 (range from2.98 to 24.13) hours for postsurgical analgesia. In this study, 0.25% ropivacaine combining with 0.8% lidocaine, at a dosage of 0.5ml/kg for a caudal block, could provide 174.3±20.5 min postsurgical analgesia. Main reasons for these differences may derive from differences in concentrations and doses of local anesthetic drugs given in caudal blocks. Moreover, this study suggests that ultrasound-guided dorsal penile nerve block can provide longer postsurgical analgesia.

In this clinical trial, changes of heart rates (HRs) and respiratory rates (RRs) were used as main analgesia indicators during surgery, because both HRs and RRs are very sensitive to inadequate analgesia, especially in children with spontaneous respiration or in the children under a low level of general anesthesia with sevoflurane 0.8 to 1.0MAC. Blood pressure was not included as an indicator in this study, mainly because noninvasive blood pressure cannot reflect real-time changes of blood pressures. In addition, it is hard to find an appropriate cuff size of blood pressure monitors to every patient. If an inappropriate cuff is applied, measured blood pressure values would deviate from actual ones, which will lead to system errors in this trial. Other postoperative indicators such as pain scores, the stay time in PACU, the first time taken to micturition, the first call for analgesia after surgeries, and the adverse effects have been used in previous relevant studies [5, 6, 15–18].

In this study, three patients in Group DPNB were excluded based on the following reasons: (i) two cases may be related to unsuccessful blocks because local anesthetics were not injected around the target nerve. This suggests that the success of the ultrasound-guided DPNB depends on a well-experienced anesthesiologist. (ii) One patient was excluded because the criteria of the study program were not followed well by one pediatric surgeon and the surgical procedure was delayed for more than 5 minutes after the regional block (protocol violations). Additionally, in Group CB, five patients were excluded due to the same reasons: three patients by protocol violations and two by the failure of the caudal block.

Some limitations were entailed in this clinical trial. Firstly, the duration of completing blocks in the two groups was not considered and compared. Due to a relative short duration of circumcision surgical procedure, the duration for a nerve block using one specific anesthesia method is one of the important indicators evaluating the efficiency of this type of operation. Despite lack of data, the time required for the two blocks in this trial is almost the same, on our experiences. Secondly, optimal dosages of local anesthetic in a specific nerve block were not detected in this study and will be confirmed in future studies. Finally, there is still a classical dorsal penile nerve block with or without ultrasound guiding, which administers local anesthetics to the base of penile. This traditional dorsal penile nerve block is also an important regional anesthesia method for pediatric circumcision surgeries. This study did not compare traditional dorsal penile nerve block with ultrasound-guided DPNB via perineal approach.

## Figures and Tables

**Table 1:** Characteristics of the patients.

Variables	group dorsal penile nerve block (n=47)	group caudal block (n=43)	*P*-value
Age(yr)	11.7±2.9	12.5±2.6	0.59
Weight(kg)	32.5±9.9	36.8±7.3	0.36
Height(cm)	156.9±15.1	161.6.1±13.2	0.45

**Table 2:** The induction time, duration of circumcision surgery, stay time in PACU, and pain score when leaving PACU between the two groups.

Variables	Group DPNB	Group CB	*P*-value
	(n=47)	(n=43)	
The induction time (sec)	142±35	153±46	0.33
The duration of circumcision surgery(incision to final stitch, min)	28.9±4.3	28.3±3.5	0.73
The staying time in PACU(min)	33.1±7.0	32.5±5.1	0.32
Pain score when leaving PACU	3.0±1.2	3.1±1.0	0.68

## Data Availability

The data used to support the findings of this study are available from the corresponding author upon request.
